# Amphibian responses in the aftermath of extreme climate events

**DOI:** 10.1038/s41598-020-60122-2

**Published:** 2020-02-25

**Authors:** Gary M. Bucciarelli, Morgan A. Clark, Katy S. Delaney, Seth P. D. Riley, H. Bradley Shaffer, Robert N. Fisher, Rodney L. Honeycutt, Lee B. Kats

**Affiliations:** 1 UCLA, Department of Ecology and Evolutionary Biology, 610 Charles E. Young Drive East, Los Angeles, CA 90095 USA; 2 La Kretz Center for California Conservation Science, 619 Charles E. Young Drive East, Los Angeles, CA 90095 USA; 3 Pepperdine University, Natural Science Division, 24255 Pacific Coast Highway, Malibu, CA 90263 USA; 4 U.S. National Park Service, 401W. Hillcrest Drive, Thousand Oaks, CA 91360 USA; 5 U.S. Geological Survey, Western Ecological Research Center, San Diego Field Station, 4165 Spruance Road, Suite 200, San Diego, CA 92101 USA

**Keywords:** Climate-change ecology, Conservation biology

## Abstract

Climate change-induced extinctions are estimated to eliminate one in six known species by the end of the century. One major factor that will contribute to these extinctions is extreme climatic events. Here, we show the ecological impacts of recent record warm air temperatures and simultaneous peak drought conditions in California. From 2008–2016, the southern populations of a wide-ranging endemic amphibian (the California newt, *Taricha torosa*) showed a 20% reduction to mean body condition and significant losses to variation in body condition linked with extreme climate deviations. However, body condition in northern populations remained relatively unaffected during this period. Range-wide population estimates of change to body condition under future climate change scenarios within the next 50 years suggest that northern populations will mirror the loss of body condition recently observed in southern populations. This change is predicated on latter 21^st^ century climate deviations that resemble recent conditions in Southern California. Thus, the ecological consequences of climate change have already occurred across the warmer, drier regions of Southern California, and our results suggest that predicted climate vulnerable regions in the more mesic northern range likely will not provide climate refuge for numerous amphibian communities.

## Introduction

The increased frequency and severity of extreme climate events make critical the need to identify vulnerable populations and spatial regions^1–5^. Because historical climate has shaped many traits that influence climate-induced vulnerability^6,7^, vulnerable populations may be identified by determining and predicting trait responses to present and projected climate^8,9^. Trait-based analyses have helped to explain species’ sensitivities to climate change^7^, but have been used less commonly as a tool to assess the impacts of recent climate extremes and to improve efforts to buffer the potential negative effects of rapid climate deviations. This is especially important across the North American Southwest that is simultaneously a biodiversity and a climate change hotspot^10^, making it at once a region faced with severe climate stress and major conservation pressure. Amphibian conservation efforts remain a global priority due to ongoing population declines^11^ and the group’s role as a suitable model to understand wide-spread biodiversity crises^12^. Here, we draw upon nearly a decade of amphibian (*T*. *torosa*) trait response data collected across the highly biodiverse California Floristic Province to quantify broadly the ecological consequences of recent extreme climatic events and to predict population responses to future climate.

Amphibians are expected to exhibit striking responses to fast-shifting climate due to characteristic traits including strong site fidelity, short dispersal and migration distances, and physiological constraints associated with ectothermy^13^. Indeed, marked departures from historical breeding phenology and geographic distributions^14^, reduced annual survival rates^15^, and lower fitness phenotypes^16^ of amphibians are all associated with recent rapid climate shifts. Amphibian reproduction is generally linked to precipitation patterns and as a result, extended drought negatively impacts population fecundity and adult survivorship^15,17^. Although amphibians have evolved life history strategies to mitigate the negative effects of variation in precipitation patterns^18^, their capacity to adapt to simultaneous drought and rapid temperature increases, as well as greater environmental variability generally, may be insufficient to maintain viable populations given the rate and intensity of such changes^19^.

**Figure 1:**
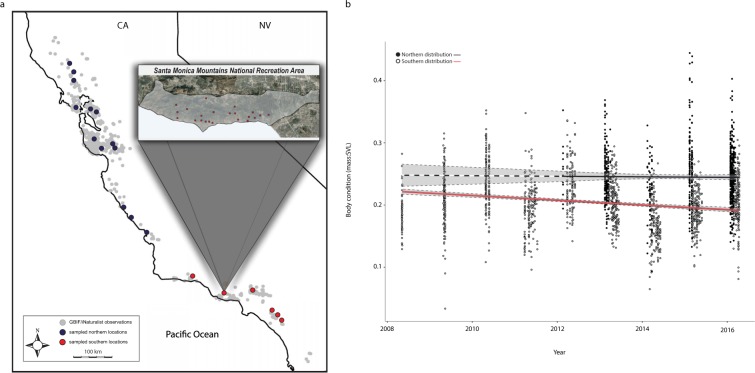
Changes to body condition across a species range. (**a**) GBIF and iNaturalist observational sites and sampled populations of a California endemic amphibian (*Taricha torosa*). The inset map depicts the spatial distribution of a focal population in the southern distribution within Santa Monica Mountains National Recreation Area (SMMNRA). (**b**) Longitudinal body condition data collected from sampled populations (northern: 2012–2016; southern: 2008–2016) highlight a significant general reduction in mean body condition (20% loss, respectively) across the southern distribution from 2008–2016 (GLMM, *F*_*1*,*25*_ = 46.45, *p* < 0.001). Grey bands surrounding trend lines show 95% confidence intervals for both distributions. The dashed segment of the trend line in the northern distribution represents extrapolated data. Maps were rendered in *R*^35^ using packages *ggplot2*^36^ and *ggmap*^37^.

To assess the ecological impacts of extreme climatic events, we evaluated adult male body condition of *T*. *torosa* from 30 sites distributed across the species’ latitudinal range (32.8°N to 39.4°N, >700 km; Fig. 1a, *sampled locations*) during a period (2008–2016) that overlapped with the most severe drought inferred to occur in California during the last 1,200 years^3^. As a trait, body condition reflects the effects of both short and longer-term environmental conditions^20^, especially prior to breeding events^21^, and as such, can impact both survivorship and fitness. Given the species’ biphasic life history and the average time required to reach sexual maturity (5 years, generally), both current environmental conditions, and conditions from metamorphosis to sexual maturity^22^ influence trait responses to climate change. We hypothesized that body condition range-wide declined during extended drought and record warm air temperatures recorded from 2012–2016 and that the impact would be most apparent in southern populations due to differences in geographic extent and duration of these events. Our evaluation of body condition across the species range indicated significant differences in trends between northern and southern distributions (Fig. 1). In general, the body condition of the southern distribution was reduced on average by nearly 20% from 2008–2016 (Fig. 1b) along with other fitness phenotypes (Supplementary Information), but we did not observe a similar trend in the northern distribution. This negative trend in the south was linked with the extent to which current and prior mean annual temperature and mean annual precipitation deviated from 20^th^-century trends (Supp. Fig. 1).

**Figure 2:**
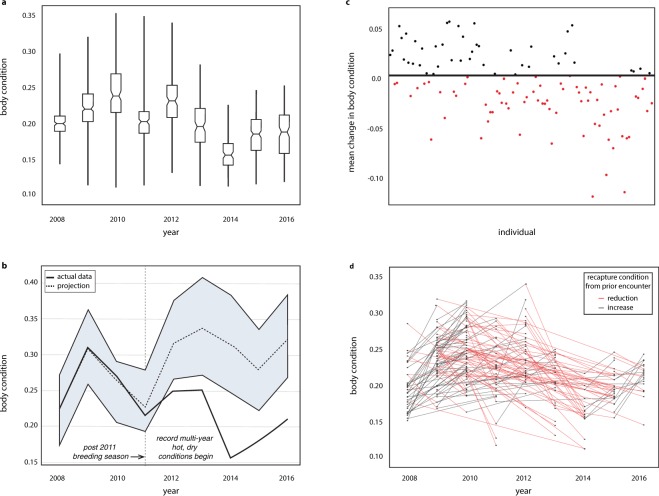
Impacts of climate change on body condition for a population and its individuals. (**a**) The SMMNRA population experienced a significant change to variance in body condition through time (*F*_1,7_ = 5.90, *P* < 0.041). Notched boxes show annual median body condition and 95% confidence intervals. Non-overlapping notched boxes suggest significant differences between medians. Whiskers extend through the range of recorded values. (**b**) Bayesian estimates of body condition differed significantly from observed values (*P* < 0.001) during peak drought and record temperatures. The blue shaded area shows the 95% confidence interval around predicted values. The solid line shows the actual data and how it deviates from estimates during record warm, dry years. Within the SMMNRA population, mean change in body condition (**c**) shows that nearly two thirds of individuals experienced a net reduction to body condition between 2008–2016. Each dot represents an individual and the overall change one experienced as either a net increase (black) or decrease (red). The pattern of loss in this population is corroborated by tracking individual recaptures. (**d**) Black points show each recapture event and lines show whether the individual increased (black) or decreased (red) body condition from prior recapture events.

To test whether the difference in body condition for the southern populations was associated with extreme climate events, we scrutinized a unique long-term dataset compiled from a mark-recapture population study in Southern California (Santa Monica Mountains National Recreation Area, Fig. 1a) to evaluate whether reduced body condition was associated with rapid increases to annual air temperature and prolonged drought beginning in 2012. We found that the total number of captured individuals and recapture success were negatively correlated with increases to mean annual air temperatures (total: *r* = −0.51; recaptures: *r* = −0.74) and with decreases to mean annual precipitation (total: *r* = −0.47; recaptures: *r* = −0.51; Supp. Fig. 2). Specifically, as mean annual temperatures and total precipitation substantially deviated from 20^th^ century trends, recapture success significantly declined (linear regression, *F*_1,7_ = 7.83, *P* < 0.027). By evaluating annual variance in body condition, we also found that body condition within this mark-recapture population was significantly related to the effects of temperature and precipitation deviations (linear regression, *F*_1,7_ = 5.90, *P* < 0.041; Fig. 2a). To further understand the impacts multi-year drought and extreme temperatures had on this population, we utilized a Bayesian time-series model^23^ trained on pre-drought (2008–2011) body condition data and climatic covariates (precipitation and temperature deviations) to predict a range of expected body condition response based on 2012–2016 precipitation and temperature deviations. Bayesian estimates of body condition from 2012 onward significantly differed from observed values (Fig. 2b). Actual population body condition fell well below the estimated ranges and at no point overlapped with pre-drought body condition estimates. In fact, cumulative impacts on body condition post-2011 were consistently negative (Supp. Fig. 3).

To develop a clearer picture of the relationship between extreme climatic events and within-population body condition dynamics, we analyzed body condition data from successively recaptured individuals. Our analysis of mean change to body condition showed that 62% of marked individuals experienced an overall reduction in body condition (Fig. 2c), and when we evaluated temporal patterns of marked individuals, we found that after 2011 body condition was primarily negative relative to the prior point a marked individual was captured (Fig. 2d). Although median body condition increases after 2014, the upper ranges remain reduced relative to earlier years (i.e. the range of boxplot whiskers), suggesting that variation in body condition within this population remains greatly depressed. This subtlety is important to note because lowered body condition is strongly associated with an increased risk of desiccation and disease, as well as decreased fecundity and adult recruitment^24^. These data confirm a rapid and significant reduction in body condition across southern populations linked with the most extreme drought and highest mean annual temperatures on record for the state^25^. During this time, reduced habitat quality in Southern California^26^ likely differentially affected southern and northern populations due to their differences in breeding phenology^27^. Northern populations tend to breed in early winter, which are typically the wettest and coolest periods of the California rain year. As a result, northern populations are possibly better buffered from recent climatic extremes than southern populations that reproduce later in the rain year when conditions are relatively warmer and drier. Collectively, these results imply that the northern populations, currently considered to be of low conservation concern, may be imperiled when regional climate mirrors recent Southern California extreme climatic events.

**Figure 3:**
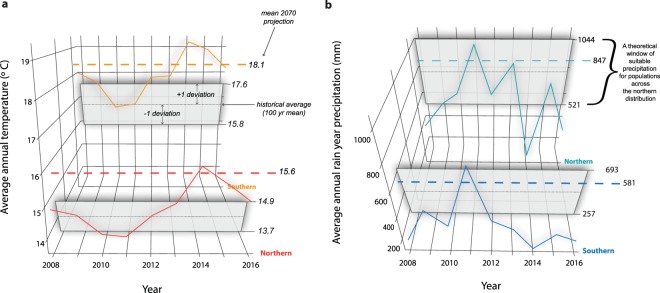
20^th^ century precipitation and temperature windows. Temperature (**a**) and precipitation (**b**) data collated from all known breeding sites throughout northern and southern distributions (z-axis) shape the climate windows for these regions. Each window is formed by a regional mean and standard deviation (±1) value calculated using PRISM annual values based on 1900–2000 data. During our study period, precipitation and temperature deviations in the southern distribution have remained outside of 20^th^ century windows since 2012. Mean 2070 predicted values are presented as dashed horizontal lines and are based on estimates from CCSM RCP 6.0 data.

**Figure 4:**
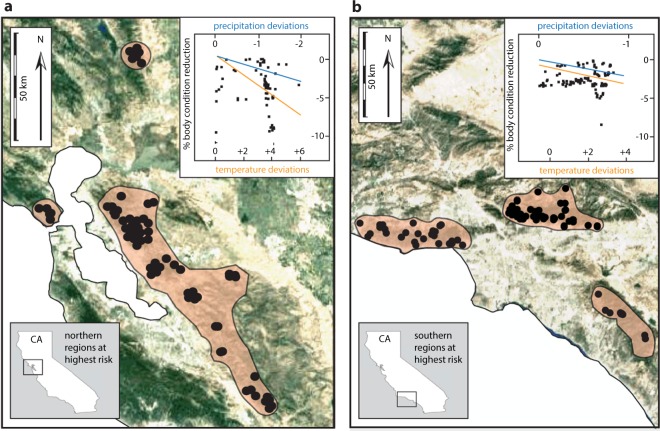
California regions at highest risk in the 21^st^ century. Inset graphs show the (**a**) northern and (**b**) southern populations predicted to experience a loss in body condition as a result of future regional climate deviations. Satellite images present the spatial distribution of these populations and the regions they occupy. Trend lines represent the magnitude of precipitation (blue) and temperature (orange) deviations driving body condition reductions. Note that the magnitude of temperature deviations in both regions is predicted to drive body condition reductions more so than precipitation deviations, especially in northern populations (**a**). Maps were rendered in *R*^35^ using packages *ggplot2*^36^ and *ggmap*^37^.

Given the links between reduced body condition in southern populations and record high air temperatures and drought, we hypothesized that similar negative responses may transpire in northern populations if precipitation and temperature patterns deviate outside of 20^th^ century climate trends over the next 50 years (Fig. 3, Supp. Fig. 4). Based on our model estimates of future body condition under predicted climate change across both the northern and southern distributions, the northern populations are predicted to exhibit the greatest response to future climate change (Fig. 4). Throughout these northern populations, the projected reduction to body condition is greatest in areas where climate models predict a nominal change from current annual precipitation (<5 cm increase) and greater departures from current mean annual temperatures (~20% increase; Fig. 4, Supp. Fig. 4). In the south, the magnitude of the response is less extreme given current precipitation and temperature patterns that are generally similar to those predicted throughout the remainder of the 21^st^ century. Because southern populations have already faced the climate of the near future, the change to body condition is less extreme relative to their northern counterpart.

Our analyses of long-term data make apparent the impacts of recent extreme climate events in California and foretell future consequences to populations. Climate for the latter part of the 21^st^ century that is predicted to remain outside of 20^th^ century climate windows (Fig. 3) will likely further contribute to negative population-level outcomes^28^. The expected shift to greater interannual variability and episodic precipitation patterns^29^ and predicted drastic increases to air temperature^30^ will likely shatter 20^th^ century climate windows. Anthropogenic habitat degradation, increased fire frequency, introduced species, and globally relevant disease dynamics further increase the climate change-induced extinction risk for many species. Currently, breeding sites of our focal southern population are found in relatively pristine habitat, largely buffered by protected land, with the highest biodiversity of benthic macroinvertebrates in the region^31^. In conjunction with over 30 years of survey data from this population, the potential alternative causes for these drastic changes, such as disease, compromised habitat, or invasive species do not explain our observed patterns. However, should these factors become relevant, we may observe geographically broader and greater cumulative ecological negative impacts. Although our findings indicate that extreme climate deviations will induce drastic trait shifts for amphibians that will likely contribute to climate change-induced population extinctions, our results also provide an early warning of the regions where researchers and land managers could develop effective conservation strategies to minimize future climate change related population losses.

## Methods

### Animal body condition data

A total of 2,323 unique individuals *(Taricha torosa*) were captured, weighed, and measured (snout-vent length, SVL) across 30 breeding sites during 2008–2016 breeding seasons (Supplementary Information). To avoid interfering with female breeding activity and egg deposition only males were captured. Individual and annual site mean body condition data were derived using a ratio of mass to SVL (Supplementary Information). Linear mixed-effects models fitted with fixed effects for geography (northern or southern) and a random effect for site were used to detect potential differences in body condition between the north and south. For these models, no random effect for individual was included because recaptures were only encountered in Santa Monica Mountains National Recreation Area and any adult that was repeatedly measured was omitted from the analyses. To generate body condition data for populations across the species range, GBIF and iNaturalist data for all known breeding sites were downloaded (n = 1,202), GPS coordinates extracted, and body condition responses predicted with models specific to each distribution (Supplementary Information). All animal-related research was approved by the University of California, Los Angeles Institutional Animal Care and Use Committee (IACUC) and conducted in accordance with relevant guidelines and regulations according to permits from IACUC (2013-011-13 C) and the California Department of Fish and Wildlife (SC-12430).

### Climate data

For all geographic points (both those from sampled populations and those from GBIF^32^ and iNaturalist observations), we acquired PRISM annual precipitation (mm) and mean annual temperature (°C) data for locations between 1900 and 2000. For sampled locations only, monthly precipitation and temperature data were also collected from October 2001 through December 2016. The 100-year annual values were used to derive century mean precipitation, century mean air temperature, and standard deviations for both. Monthly data (2001–2016) for temperature (Jan.–Dec.) and precipitation (water-year; Oct.–Sept.) were used to derive mean annual temperature and cumulative water-year total precipitation at the sites we sampled, and these values served as predictors in our analyses. Mean century precipitation and temperature data from all sites in northern and southern distributions were used to calculate a mean temperature and precipitation value in each distribution for years 2008–2016. Rasterized *Worldclim 1*.*4* mean 2070 temperature (bio1) and annual precipitation (bio12) layers (CCSM4, RCP 6.0) were used to extract projected climate values for all GBIF/iNaturalist sites across California (Supplementary Information). Finally, *Worldclim 1*.*4* current climate data were rasterized to visualize the degree of difference in precipitation and temperatures over the species’ distributional range.

### Climate deviations from century trends

To calculate the extent an annual value (2001–2016) of precipitation or temperature deviated from the century average (*deviation value*), we performed a *z*-score calculation and subtracted the annual observed value from the century average then divided the difference by the deviation value ((observed value – century mean)/century standard deviation). Deviation values were used for statistical analyses.

### Analyses of body condition

To determine if trends in body condition from sampled sites were linked with climate patterns leading up to and through drought and severe temperatures (2008–2016), we used *randomForest*^33^ in *R* to construct classification and regression tree models for southern and northern distributions independently and tested whether annual mean temperature and precipitation deviations for the current year of sampling and annual periods dating back six years (to capture time to sexual maturity) explained changes to body condition (Supplementary Information). Model results were permuted with 1,000 iterations to derive p-values and identify significant predictors of body condition.

### Estimated current and future trait responses

Initial models for northern and southern distributions were updated to include only significant climate predictors, cross-validated with separate training and test datasets, and accuracy assessed with observed versus predicted model results (northern: *R*^2^ = 0.79, southern: *R*^2^ = 0.74). To assess potential species-level effects of extreme climate, models and associated covariates were parameterized with observed deviation values to predict current body condition in all non-sampled (GBIF/iNaturalist) sites separately in the northern (n = 864) and southern (n = 319) distributions. Future body condition was estimated similarly, but with extracted 2070 *Worldclim 1*.*4* data. To predict ultimate body condition outcomes, we used the model results of current and future body condition values from each non-sampled site to derive a percent change in body condition by 2070. We then mapped only those sites predicted to experience body condition loss (i.e. our predicted at-risk populations, highlighted by colored polygons on the satellite images) and used this dataset to evaluate the overall trend in change to body condition as a function of 2070 precipitation and temperature deviations.

### Empirical population and individual level responses

Population level responses to climate change were assessed using body condition data from males collected during 2008–2016 breeding periods in a mark-recapture population^34^ (Santa Monica Mountains Recreation Area, Los Angeles, CA, USA). For each year, we calculated total individuals captured (unmarked and marked; range of annual sample sizes: *unmarked* = 110–210; *marked* = 24–89, see Supplementary Information), mean body condition of the population, total years marked individuals were recaptured, and recapture success, which we calculated as percent of total marked individuals recaptured, standardized by cumulative total marked to account for increasing effort each year. Data were evaluated relative to current temperature and precipitation patterns and century trends. Individual body condition trends and the cumulative impact of drought on body condition were tested using a Bayesian time-series framework within the *CausalImpact* package^23^ for *R* to assess whether changes to body condition within individuals significantly differed from projected patterns due to climatic extremes.

## Supplementary information

Supporting Information.
