# Supplementary material for: Amphibian responses in the aftermath of extreme climate events
Source: Sci Rep. 2020 Feb 25;10:3409. doi: 10.1038/s41598-020-60122-2 (PMC7042276; doi:10.1038/s41598-020-60122-2)
Supplement: Supplementary file 1 — Supporting Information. [file 41598_2020_60122_MOESM1_ESM.docx]

**Supplementary Information**

*Amphibian responses in the aftermath of extreme climate events*

Gary M. Bucciarelli ^1, 2, *^, Morgan Clark ^3^, Katy S. Delaney ^4^, Seth P.D. Riley ^4^, H. Bradley Shaffer ^1, 2^, Robert N. Fisher ^5^, Rodney L. Honeycutt ^3^, Lee B. Kats ^3^

^1^ *UCLA, Department of Ecology and Evolutionary Biology*

*610 Charles E. Young Drive East*

*Los Angeles, CA 90095*

^2^ *La Kretz Center for California Conservation Science*

*619 Charles E. Young Drive East*

*Los Angeles, CA 90095*

^3^ *Pepperdine University, Natural Science Division*

*24255 Pacific Coast Highway*

*Malibu, CA 90263*

^4^ *U.S. National Park Service*

*401 W. Hillcrest Drive*

*Thousand Oaks, CA 91360*

^5^ *U.S. Geological Survey, San Diego Field Station*

*4165 Spruance Road, Suite 200*

*San Diego, CA 92101*

*Correspondence garyb@ucla.edu

**
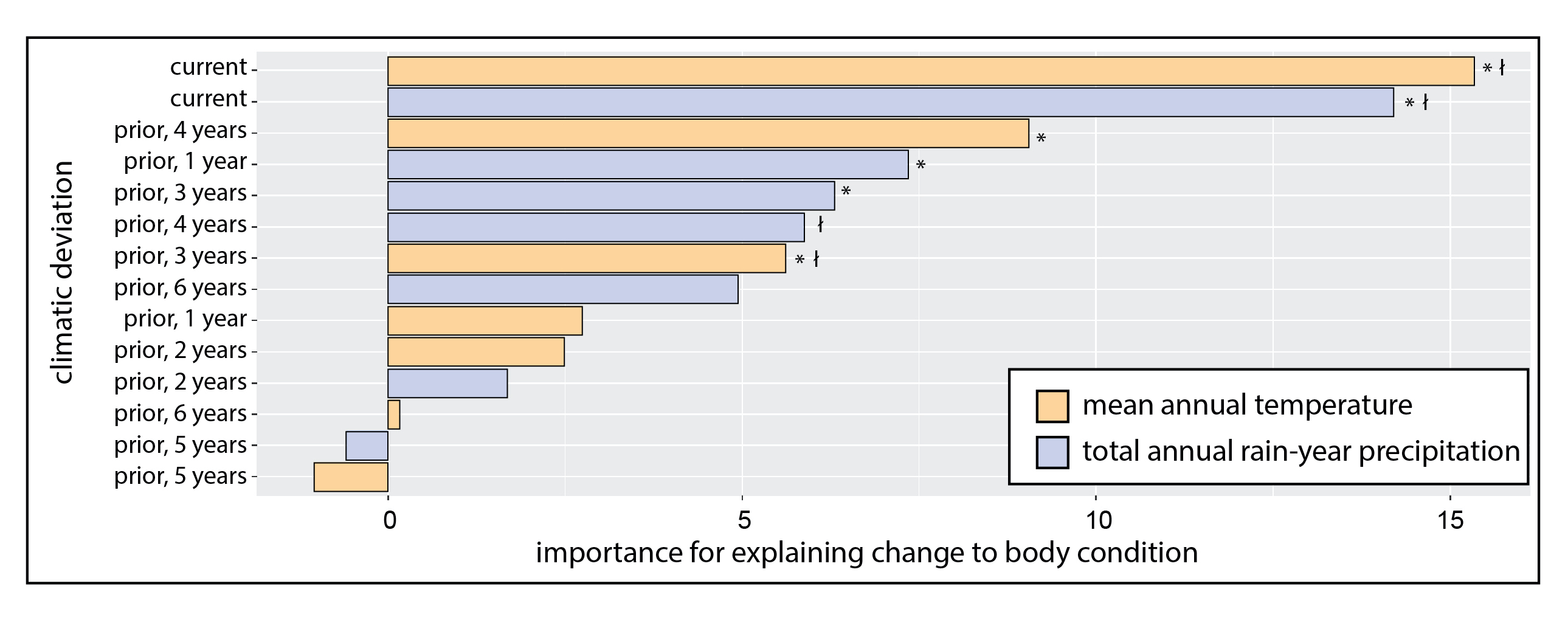
**

Supp. Fig. 1. **Current and prior precipitation and temperature predictors explain changes in southern newt body condition.** Variables from the *randomForest* model indicate current and prior deviations in temperature and precipitation explain body condition in southern populations. Importance is computed as the relative loss in predictive performance of the model after random reordering values of each predictor. Glyphs indicate the significant predictors for southern (*) or northern () deviation values of total annual rain-year precipitation and mean annual temperature that explain changes in body condition. Significance based on permutation tests (p < 0.05).


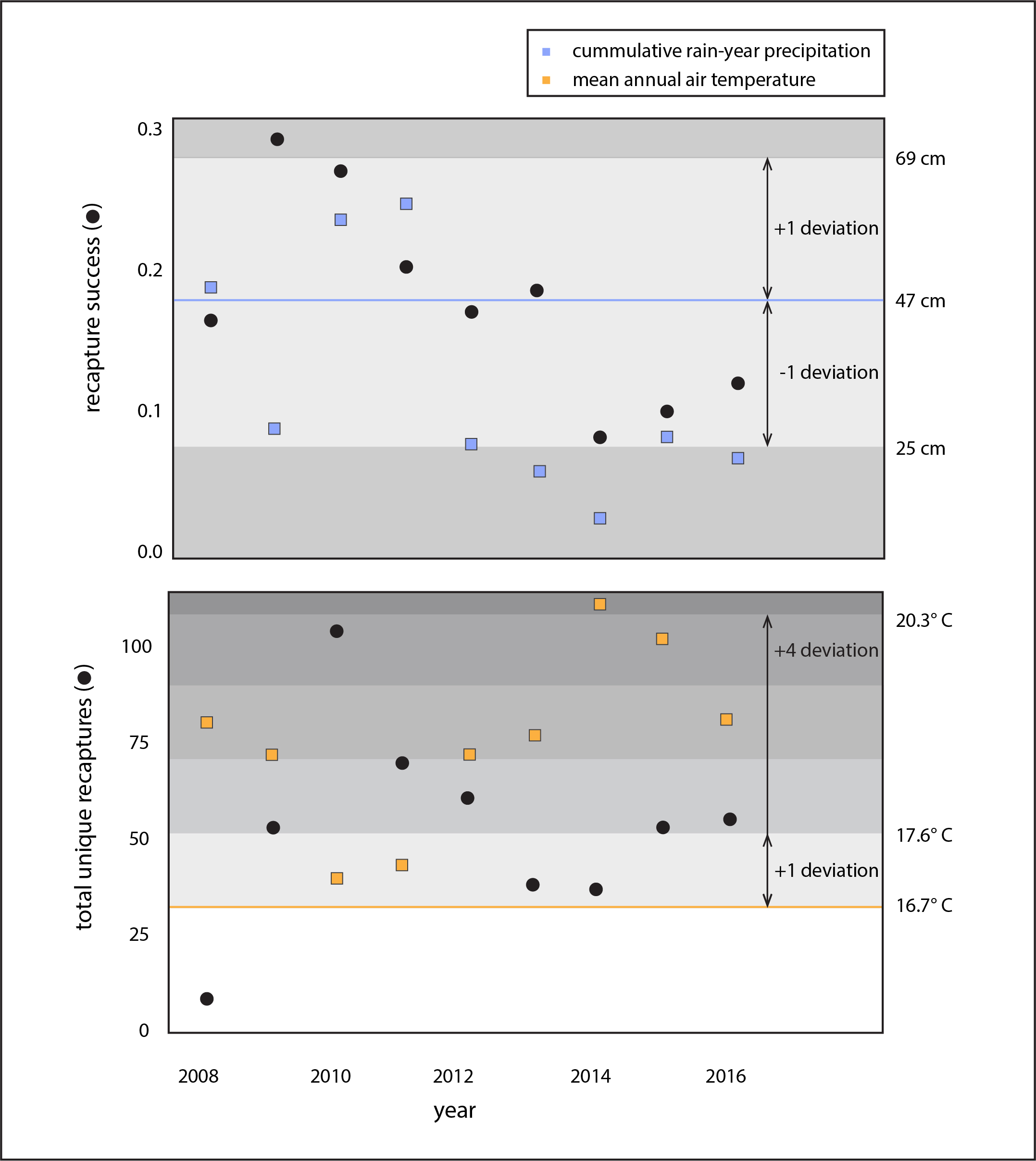


Supp. Fig. 2. **The** **population dynamics of a Southern California capture-mark-recapture population in response to extreme climatic events.** (a) Recapture success of marked individuals and (b) number of unique marked newts annually encountered from surveys in conjunction with annual total precipitation (upper panel) and mean annual temperatures (lower panel). The 20^th^ century mean (solid colored horizontal line) for each climatic variable and associated deviation values are superimposed in the background.


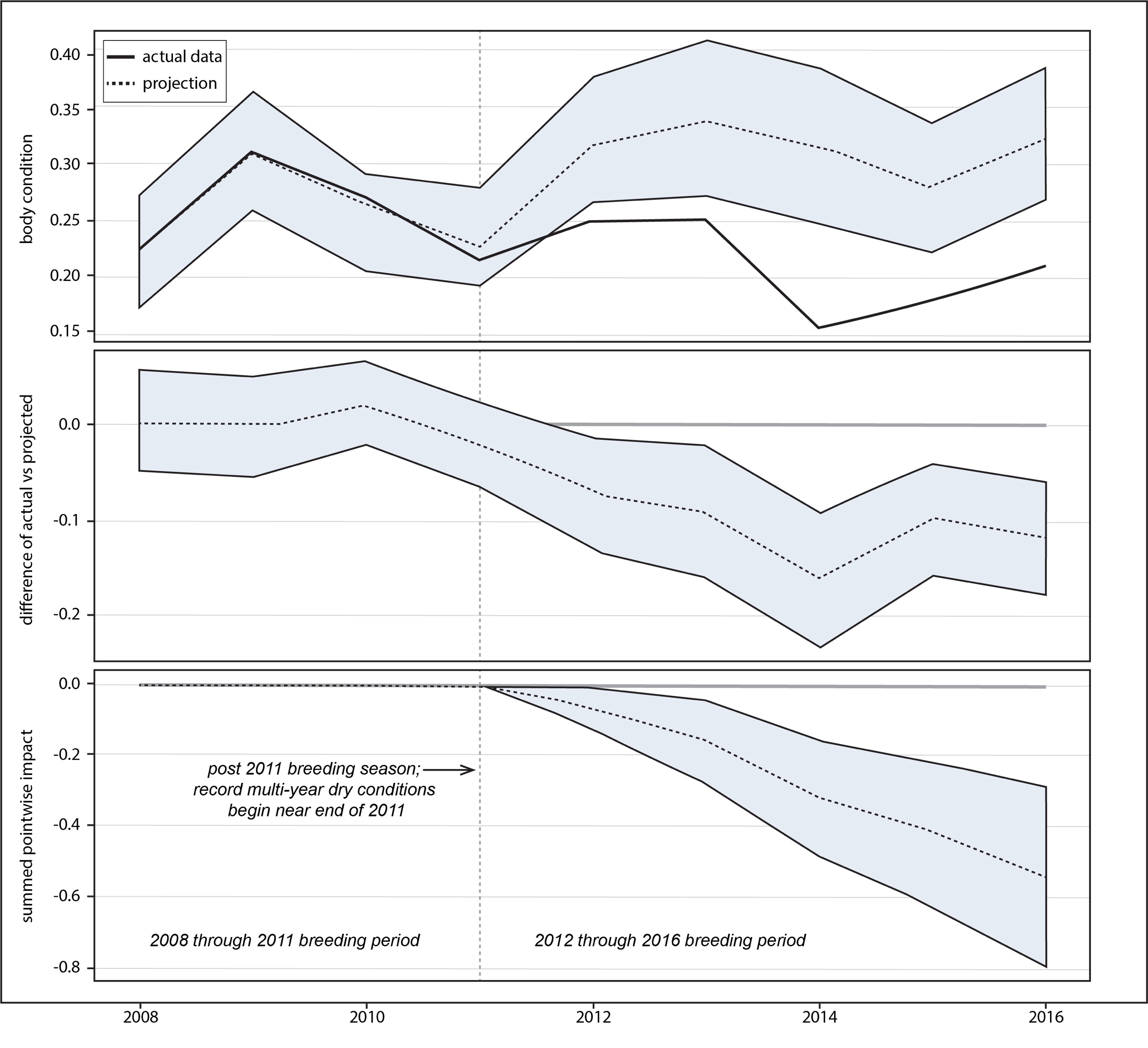


Supp. Fig. 3. **Bayesian estimates of changes to body condition in Southern California populations from 2008-2016**. The top panel shows actual and projected body condition following the onset of extreme conditions, with pointwise differences from projected values of each year in the middle panel. The bottom panel shows the cumulative change in body condition, highlighting the overall net loss as drought progressed and record-setting warm air temperatures occurred.


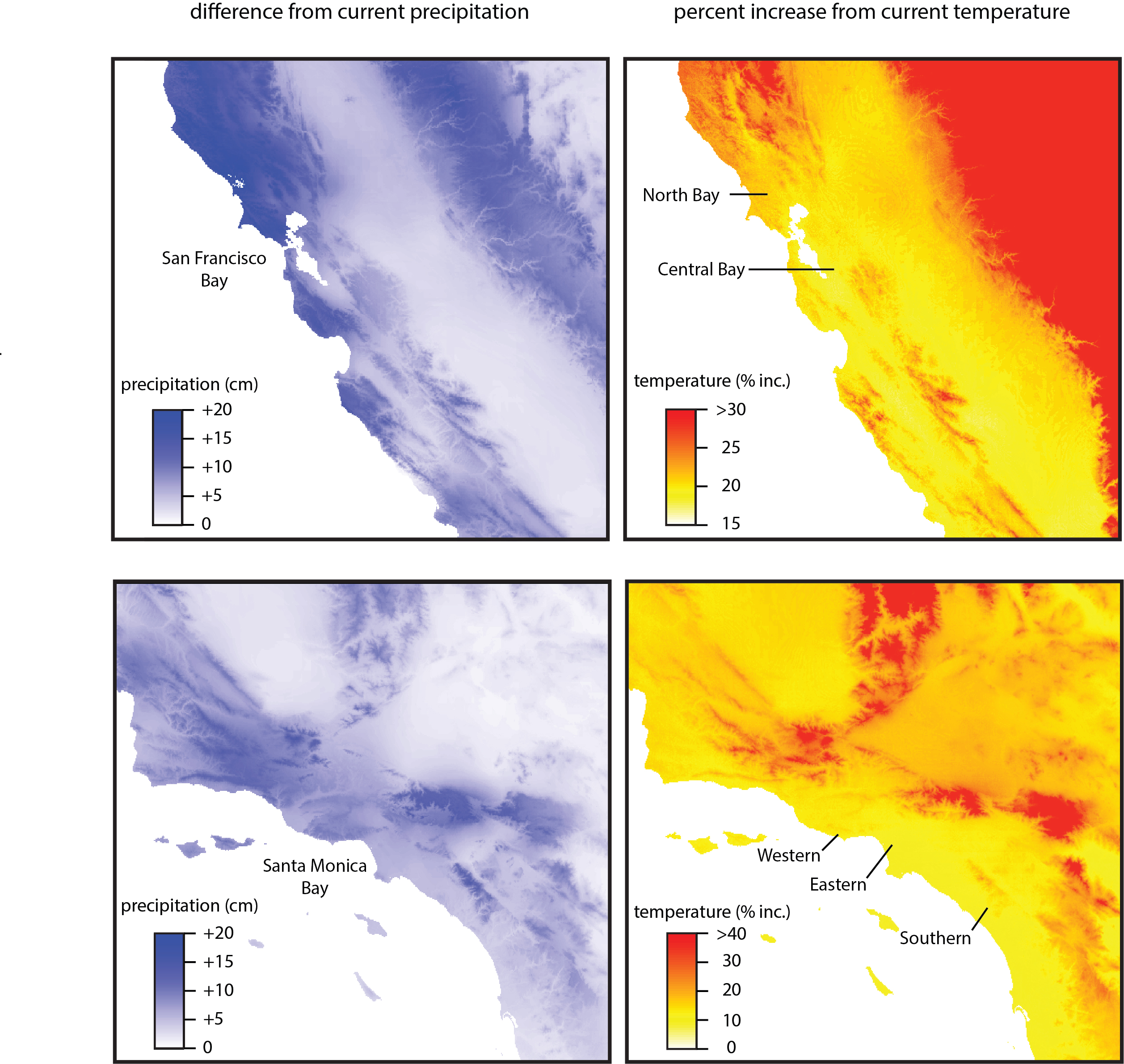


Supp. Fig. 4. **Projected change in annual total rain-year precipitation and annual mean air temperature by 2070 relative to climate during the 20^th^ century**. Marked climate differences are apparent across both the northern (upper panels) and southern (lower panels) distributions. Habitat across the northern distribution throughout the North and Central Bay Areas supports a vast majority of remaining breeding populations in all of California (Fig. 1). The western and eastern extent of the Santa Monica Bay and southern Santa Ana Mountains are the areas that support the greatest number of remaining populations throughout Southern California. Note the difference in scale between percent increase of temperature between the northern and southern locations.

**SUPPORTING METHODS:**

*Animal body condition data*: Animals were collected and marked following methods in Watters and Kats (2006) whereby a passive integrated transponder is interperitoneally injected within the abdomen. To calculate body condition, we used a ratio of mass to snout-vent length (mass:SVL) because we only made comparisons between adult males, which obviated the need to account for ontogenetic effects (Peig and Green 2010).

We delineated northern and southern distributions based on the greatest geographical gap between populations across Point Conception and the Tehachapi Mountains (Thomson et al. 2016). Linear mixed-effects models were created in *R* (*R* core team) using the *lme4* package (*v. 1.1-20*, Bates et al. 2014). Body condition was regressed against a categorical predictor for distribution (southern or northern) with a random effect for site to account for repeated sampling and potential differences between sites.

The package *rgbif* (*v. 1.1.0*, Chamberlain et al. 2017) in *R* was used to download GBIF and iNaturalist observational data via the *name_backbone* and *occ_search* functions. Data were cross-checked for accuracy to ensure that data points fell within the species’ recognized range. Any GPS coordinates that mapped off-land (i.e. to marine habitat, often due to masking) were removed, leaving 1,202 locations.

*Climatic data*: PRISM data were accessed and downloaded via prism.oregonstate.edu. *Worldclim 1.4* GCM CCSM4 layers were accessed and downloaded via worldclim.org (Hijmans et al. 2005). Given current trends that actually track more closely to RCP 8.5, we chose to utilize RCP 6.0 as a conservative estimate of carbon emissions over the next 50 years. We utilized *Worldclim 1.4* climate data layers (1960-1990) to visualize degree of difference between current and 2070 climate because alternative data layers (1970-2014) included current climate change data.

*Analyses of body condition*: The northern and southern species-level models in *randomForest* treated mean annual body condition from each site as the response with precipitation and temperature deviation values as predictors. Prior years were coded relative to a current sampling period. Up to six years (i.e. six years of annual climate data that occurred prior to a year) were included to capture potential amphibian life-history trade-offs associated with poor habitat during development (Searcy et al. 2014). Because classification and regression tree type models do not return traditional test statistics (i.e. p-values), we used the *rfPermute* package to identify statistically significant climatic variables in the northern or southern distribution models, then updated these models to include only those significant variables for predictions. The updated northern model included current temperature and precipitation deviation values, prior precipitation deviation values that included both a single year and 4 years prior to the relative year of sampling, and annual temperature deviations 3 years prior to the relative year of sampling. The southern model included current temperature and precipitation deviation values, 1 and 3 three years prior precipitation deviation values, and 3 and 4 year prior temperature deviation values. To estimate current body condition across sites from GBIF and iNaturalist sites, we generated independent data frames for each distributional range based on measured minimum and maximum values from each significant predictor in each range and predicted body condition from these distributions. To predict future body condition for each site from GBIF/iNaturalist observations, we followed similar procedures used to predict current body condition, but updated models with only estimated 2070 mean annual temperature or total annual precipitation deviation values. Each independent data frame was iterated 10,000 then averaged to generate future body condition data.

To estimate ultimate outcomes, percent change in body condition for each site was calculated and compared to predicted temperature and precipitation deviations. We then mapped populations expected to experience losses to body condition due to negative deviations from century means (higher temperatures or reduced precipitation).

*Analyses of additional fitness-associated traits:* Our evaluation of additional fitness-associated traits focused on snout-vent length (SVL) throughout our study. We regressed body length through time (years) allowing for inter-site variation with a random effect for each breeding population. The results of this analysis indicated a significant difference in SVL from 2008 through 2016 (linear regression, *F_1, 1471_* = 4.17, *P* = 0.041). Overall, we detected a reduction in mean SVL measurements throughout the southern distribution from 2008 through 2016 (2018: 70.35 mm (± 0.67); 2016: 66.70 mm (± 0.35)).

| **Year** | **SMMNRA Pop.** | **Marked Pop.** |
| --- | --- | --- |
| 2008 | 139 | 40 |
| 2009 | 210 | 80 |
| 2010 | 168 | 89 |
| 2011 | 182 | 44 |
| 2012 | 135 | 46 |
| 2013 | 110 | 27 |
| 2014 | 110 | 24 |
| 2015 | 191 | 37 |
| 2016 | 130 | 25 |

Supp. Table 1. Sample sizes of individuals measured in the SMMNRA population and the focal mark-recpature population from 2008-2016.

**SUPPORTING METHODS**

**Literature Cited**

Bates D, Maechler M, Bolker B, Walker S (2014). lme4: Linear Mixed-Effects Models Using Eigen and S4. R package version 1.1-7.

Brodersen KH, Gallusser F, Koehler J, Remy N, & Scott SL. (2015) Inferring causal impact using Bayesian structural time-series models. Annals of Applied Statistics. 20159:247-274.

Chamberlain, S., Ram, K., Barve, V., Mcglinn, D. and Chamberlain, M.S., 2017. Package ‘rgbif’.

Hijmans, RJ, Cameron, SE, Parra, JL, Jones, PG, & Jarvis, A. (2005) Very high resolution interpolated climate surfaces for global land areas. International Journal of Climatology. 25:1965-1978.

Peig, J, & Green AJ. (2010) The paradigm of body condition: a critical reappraisal of current methods based on mass and length. Functional Ecology. 24:1323-1332.

R Core Team (2015). R: A Language and Environment for Statistical Computing. R Foundation for Statistical Computing, Vienna, Austria.

Watters TS, & Kats L. 2006 Longevity and Breeding Pool Fidelity in the California Newt (Taricha torosa): A Long-term Study using PIT Tagging. Herpetological Review 37:151-152.
